# Effects of Virtual Reality–Based Interventions for Promoting Physical Activity in Patients With Heart Failure: Systematic Review

**DOI:** 10.2196/86567

**Published:** 2026-03-24

**Authors:** Jeong-Ah Ahn, Jung Eun Lee, Kyoung-A Kim

**Affiliations:** 1 College of Nursing and Research Institute of Nursing Science, Ajou University, Suwon, Republic of Korea; 2 College of Nursing, University of Rhode Island, Kingston, RI, United States; 3 Suwon Women’s University, 72, Onjeong-ro, Gwonseon-gu, Suwon, 16632, Republic of Korea, 82 31-290-8240

**Keywords:** virtual reality, heart failure, physical activity, systematic review, rehabilitation

## Abstract

**Background:**

Heart failure (HF) is a progressive chronic condition associated with reduced physical and functional capacity, psychological burden, cognitive decline, and diminished quality of life (QOL). Although exercise-based cardiac rehabilitation is beneficial, participation remains low due to accessibility, physical constraints, and motivational barriers. Virtual reality (VR)–based interventions, including immersive platforms and exergaming, may enhance accessibility and engagement and promote physical activity through interactive experiences. However, evidence regarding their effectiveness in patients with HF remains fragmented.

**Objective:**

This systematic review synthesized current evidence on the effects of VR-based interventions on physical activity, psychosocial outcomes, and self-management behaviors in patients with HF.

**Methods:**

We systematically searched PubMed, CINAHL, Embase, and Scopus for studies published within the past 10 years. Randomized controlled trials (RCTs) and non-RCT interventional studies involving adults with HF who participated in VR-based interventions were eligible. Outcomes included physical activity or exercise capacity, psychological well-being, self-management, and QOL. The reviewers screened articles, extracted data, and assessed risk of bias using version 2 of the Cochrane risk-of-bias tool for randomized trials for RCTs and the Risk of Bias in Nonrandomized Studies of Interventions tool for non-RCTs. The review adhered to the PRISMA (Preferred Reporting Items for Systematic Reviews and Meta-Analyses) 2020 and PRISMA-S (PRISMA extension for reporting literature searches) guidelines.

**Results:**

A total of 10 studies met the inclusion criteria, comprising 7 (70%) RCTs and 3 (30%) non-RCTs. Studies were conducted across multiple countries and predominantly included older adults (mean age ≥65 years). Most interventions were home based, with exergaming as the most frequent modality, followed by immersive VR cycling and digital coaching programs, delivered over 4 to 12 weeks. Across studies, VR-based interventions were associated with improvements in exercise capacity (n=6, 60% of the studies), physical activity (n=5, 50%), and QOL (n=4, 40%). Three of the studies (30%) reported reductions in depressive symptoms, whereas effects on anxiety and self-efficacy were inconsistent. Adherence and usability were high across studies, and no intervention-related adverse events were reported. However, the risk of bias was rated as “some concerns” or “high” in several domains, and heterogeneity in intervention design and outcome measurement, along with small samples, limited pooled synthesis and overall certainty of evidence.

**Conclusions:**

VR-based interventions show promise as accessible and engaging approaches to promote physical activity and support rehabilitation in patients with HF, particularly in home-based settings. Across the included studies, VR interventions were generally associated with improvements in exercise capacity, physical activity, QOL, and depressive symptoms, with high adherence and no reported safety concerns. However, interpretation is limited by heterogeneity in intervention design, small sample sizes, and methodological constraints. Future research should prioritize larger, rigorously designed trials to support sustained clinical impact.

## Introduction

Heart failure (HF) is a chronic, progressive condition that affects millions of people worldwide, imposing a substantial burden on individuals, families, and health care systems. As the final pathway of various cardiovascular diseases, the prevalence of HF continues to rise due to advances in cardiac care that have improved survival rates, along with the global aging population [1,2]. HF is associated not only with physiological decline but also with multidimensional challenges that impact patients’ psychological, cognitive, and social well-being [1]. Common symptoms such as fatigue, dyspnea, and exercise intolerance limit physical activity and may lead to deconditioning, reduced mobility, and loss of independence [3]. In addition, depression, anxiety, cognitive impairment, and diminished quality of life (QOL) are highly prevalent and further complicate disease management and self-care [4,5].

Maintaining adequate physical activity is an essential component of HF management and rehabilitation. Evidence consistently indicates that regular exercise improves exercise tolerance, muscle strength, endothelial function, and overall cardiovascular health [6]. Moreover, physical activity has positive effects on psychological outcomes such as mood, motivation, and perceived QOL. However, many patients with HF remain inactive due to fear of symptom exacerbation, lack of motivation, comorbidities, and limited access to supervised rehabilitation programs [7]. Although traditional cardiac rehabilitation (CR) is effective, participation rates remain low because of transportation barriers, cost and time burdens, and reduced self-efficacy [8,9]. Therefore, innovative and patient-centered approaches are needed to enhance participation and promote sustainable physical activity among individuals with HF.

In this context, virtual reality (VR) has emerged as a promising technological tool for health promotion and disease management. VR refers to computer-generated, interactive, 3D environments that simulate real or imagined scenarios, allowing users to engage in immersive experiences [10]. These environments can elicit strong sensory and emotional engagement, making activities more enjoyable, motivating, and meaningful. The immersive nature of VR creates a sense of presence—an illusion of “being there”—which enhances focus, reduces distraction, and increases adherence to therapeutic exercises [11]. In health care, VR has been applied in various fields, including pain management, mental health therapy, motor rehabilitation, and chronic disease management [12-15]. Advances in hardware and software have made VR systems increasingly accessible, portable, and cost-effective, expanding their potential for clinical and home-based rehabilitation.

VR-based interventions have demonstrated positive outcomes across diverse patient populations. In neurological and musculoskeletal rehabilitation, VR programs have been shown to improve balance, motor coordination, and motivation [16-18]. In patients with chronic conditions such as stroke, Parkinson disease, and chronic obstructive pulmonary disease, VR-based exercise interventions have contributed to enhanced physical performance and QOL [19,20]. Moreover, VR provides a safe and controlled environment for graded physical activity tailored to patients’ physical capacities and psychological needs. The real-time feedback and gamified nature of VR exercises can foster self-efficacy and promote behavior change [21]. These features are particularly valuable for patients with HF, who often face both physical limitations and psychological barriers to exercise participation. Therefore, VR-based rehabilitation may represent a paradigm shift from conventional, clinic-based rehabilitation programs toward more interactive and personalized care.

Despite growing evidence supporting the benefits of VR-based interventions, their application in HF populations remains relatively limited. Therefore, a comprehensive review of current evidence is required to understand both the potential and the limitations of VR in this population. Given the multifaceted nature of HF and the capacity of VR-based programs to improve physical activity, a systematic review on this topic has significant clinical implications. Evaluating the effects of VR interventions on physical activity and related outcomes among patients with HF can help clarify whether VR serves as an effective adjunct or alternative to traditional rehabilitation. Furthermore, identifying the types of VR interventions, their key components, and outcome measures can inform best practices for future program design.

Therefore, this systematic review aimed to synthesize current evidence on the effects of VR-based interventions for promoting physical activity, psychosocial outcomes, and self-management behaviors in patients with HF. By synthesizing and critically appraising existing evidence, this study aimed to establish a scientific foundation for incorporating VR technologies into chronic disease self-management strategies for patients with HF.

## Methods

### Search Strategy

This systematic review was conducted in accordance with the PRISMA (Preferred Reporting Items for Systematic Reviews and Meta-Analyses) [22] and PRISMA-S (PRISMA extension for reporting literature searches) guidelines [23]. A comprehensive search was conducted in 4 electronic databases: PubMed, CINAHL, Embase, and Scopus. Each database was searched individually on its own platform (PubMed via the National Center for Biotechnology Information, CINAHL via EBSCOhost, Embase via Elsevier, and Scopus via Elsevier); no simultaneous multidatabase searching on a single platform was conducted. The search covered the period from January 1, 2016, to December 31, 2025, and the final search was completed on January 2, 2026. All retrieved articles were compiled, and duplicates were removed. The bibliographic management software EndNote (version 21; Clarivate Analytics) was used to manage and organize references.

The search terms were developed based on the population, intervention, comparison, and outcomes framework: “heart failure” (population); “virtual reality,” “immersive virtual reality,” “augmented reality,” “mixed reality,” “exergaming,” or “exergame” (intervention); “standard care” or “traditional exercise programs” (comparison); and “physical activity,” “exercise capacity,” “psychological and cognitive outcomes,” “self-management behaviors,” and “quality of life” (outcomes), including relevant synonyms and related terms. Search terms were combined using Boolean operators (“AND” and “OR”) and adapted to each database’s indexing system. Both MeSH (Medical Subject Headings) and free-text keywords were used to ensure a comprehensive search. The search strategy was further expanded to include a wider range of controlled vocabulary terms and text words related to HF (eg, “cardiac failure” and “cardiac insufficiency”), virtual and extended reality technologies (eg, “virtual reality exposure therapy,” “mixed reality,” “smart glasses,” and “head-mounted display”), exergaming and active video gaming, and physical activity–related outcomes (eg, “exercise” and “fitness”). The search strategy applied limits to publication year (past 10 years) and language (English only). The publication period was limited to the last 10 years to capture current evidence. The full reproducible search strategies for each database are provided in Multimedia Appendix 1 in accordance with PRISMA-S recommendations. The search strategy was peer reviewed by an independent librarian to ensure comprehensiveness and accuracy. Two reviewers independently conducted the database searches and verified the search outputs.

The inclusion criteria were (1) studies involving patients with HF, (2) VR-based interventional studies, (3) randomized controlled trials (RCTs) or non-RCTs, and (4) articles published in peer-reviewed journals. Eligible studies were required to include at least one outcome related to physical activity (eg, exercise capacity, activity level, or performance), whereas additional outcomes such as psychological status, self-management behaviors, or QOL were also considered. Studies that did not aim to promote or measure physical activity in patients with HF were excluded. Exclusion criteria included reviews, protocols, conference abstracts, noninterventional studies, and studies that did not report physical activity or related outcomes, as well as those lacking VR components.

Two independent reviewers conducted the screening and selection process. Any discrepancies regarding study inclusion or exclusion were resolved through discussion and consensus. When consensus could not be reached, a third reviewer was consulted. Data were extracted for all outcomes related to physical activity, exercise capacity, psychosocial status, self-management behaviors, and QOL across all reported measures and time points in each study. Physical activity–related outcomes were considered primary outcomes for synthesis and interpretation, while psychosocial and self-management outcomes were treated as secondary outcomes.

In addition to outcome data, information on study characteristics (country and study design), participant characteristics (sample size, age, and sex), and intervention characteristics (type, duration, frequency, and setting) was extracted using a predefined data extraction form. When information was missing or unclear, data were reported as not available, and no assumptions or imputations were made; study authors were not contacted for clarification.

### Risk-of-Bias Assessment

The methodological quality and risk of bias of the included studies were evaluated using standardized tools appropriate to each study design. For RCTs, version 2 of the Cochrane risk-of-bias tool for randomized trials was used [24]. This tool assesses five domains of potential bias: (1) the randomization process, (2) deviations from intended interventions, (3) missing outcome data, (4) measurement of outcomes, and (5) selection of the reported results. For nonrandomized studies, the Risk of Bias in Nonrandomized Studies of Interventions tool was used [25]. The Risk of Bias in Nonrandomized Studies of Interventions evaluates seven domains: (1) confounding, (2) selection of participants, (3) classification of interventions, (4) deviations from intended interventions, (5) missing data, (6) measurement of outcomes, and (7) selection of the reported results.

Two reviewers independently assessed each study using these tools. Any disagreements were resolved through discussion or, when necessary, consultation with a third reviewer. The overall risk of bias was categorized into 3 levels: low risk, some concerns or moderate, or high or serious risk.

### Synthesis Methods

Studies included in the narrative synthesis met the inclusion criteria and reported outcomes related to physical activity or exercise capacity in patients with HF. Studies were grouped by intervention type, study setting, and outcome domain and synthesized descriptively using reported data without conversion or imputation; meta-analysis was not conducted due to heterogeneity across studies, and potential sources of heterogeneity were explored descriptively.

## Results

### General Characteristics and Research Methodology of the Included Studies

**Figure 1.**
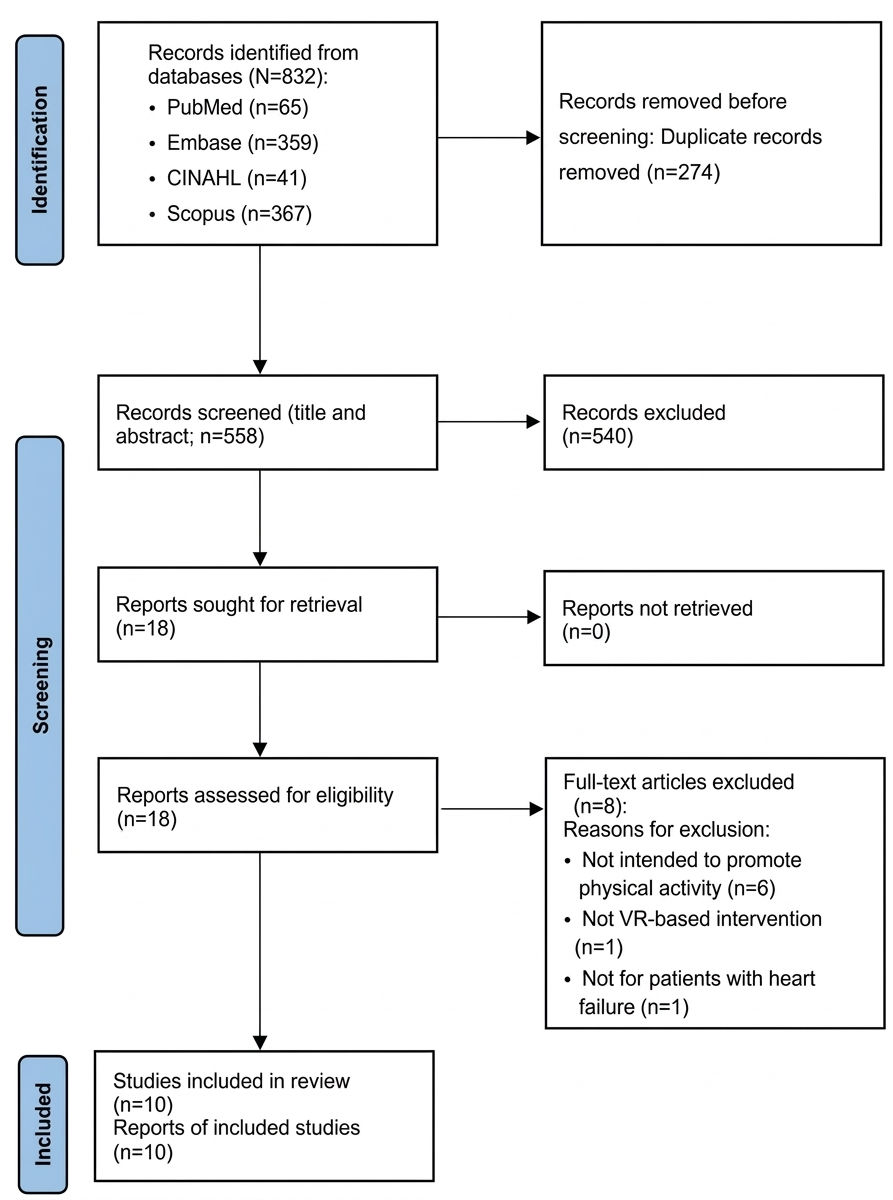
PRISMA (Preferred Reporting Items for Systematic Reviews and Meta-Analyses) flow diagram of the study selection process. VR: virtual reality.

A PRISMA flow diagram (Figure 1) was used to illustrate the study selection process. A total of 832 records were identified through PubMed, CINAHL, Embase, and Scopus. Of these 832 records, after removing 274 (32.9%) duplicates, 558 (67.1%) studies remained for title and abstract screening. Of these 558 articles, 540 (96.8%) articles were excluded for not meeting the inclusion criteria. The full texts of 18 studies were then reviewed, and 8 (44.4%) were excluded due to ineligible populations, interventions, or outcome reporting—specifically, studies aiming to improve knowledge and self-care, to manage pain, to compare hemodynamic parameters, to confirm system usability and satisfaction, or to assess symptom experience—resulting in a final selection of 10 (55.6%) articles. These comprised 7 RCTs and 3 nonrandomized single-arm studies. Table 1 and Multimedia Appendix 2 [26-35] summarize the general characteristics and research methodologies of the included studies. The studies were conducted in various countries, including Sweden, the United States, Germany, Israel, the Netherlands, Brazil, Italy, Romania, and Spain. Sample sizes ranged from 10 to 605 participants, and most studies (6/10, 60%) included older adults, with a mean age of ≥65 years. In total, 80% (n=8) of the interventions were implemented in home-based settings, whereas 20% (n=2) were conducted in hospital environments.

**Table 1.** Characteristics of the included studies (N=10).

Variable and category	Studies, n (%)
Disease of participants	
Heart failure	10 (100)
Country^a^	
Sweden	5 (50)
United States	4 (40)
Germany	3 (30)
Israel	3 (30)
The Netherlands	3 (30)
Brazil	2 (20)
Italy	2 (20)
Others (Romania and Spain)	2 (20)
Mean age of participants (y)	
<65	3 (30)
≥65	6 (60)
Not mentioned	1 (10)
Sample size	
<50	4 (40)
50-100	3 (30)
>100	3 (30)
Use of theoretical framework	
Yes	1 (10)
No	9 (90)
Study design	
Randomized controlled trial	7 (70)
1-group (single-arm) study	3 (30)
Setting	
Home	8 (80)
Hospital	2 (20)
Type of intervention	
Exergame	6 (60)
Immersive VR^b^-assisted cycle ergometer	2 (20)
Sensor-controlled digital game	1 (10)
Virtual application for cardiac rehabilitation	1 (10)
Intervention period	
During hospitalization	2 (20)
4 wk	2 (20)
12 wk	6 (60)
Follow-up frequency	
Once	7 (70)
3 times	3 (30)

a Some studies were conducted across multiple countries simultaneously. Therefore, the total number of countries listed exceeds the total number of included studies (N=10) as each multinational study was counted once per country.

b VR: virtual reality.

Only 10% (1/10) of the studies [29] applied a theoretical framework—the Fogg behavior model—to guide intervention design, whereas the remaining studies were empirically developed. Intervention durations varied, with most (6/10, 60%) lasting 12 weeks, 20% (2/10) lasting 4 weeks, and 20% (2/10) being conducted during hospitalization. The frequency of follow-up assessments ranged from a single posttest to up to 3 follow-up evaluations.

Regarding the types of VR-based interventions, exergaming was the most common approach, including Nintendo Wii–based programs (5/10, 50%) and a mobile exergame (Heart Farming; 1/10, 10%), followed by immersive VR-assisted cycle ergometer training (2/10, 20%), sensor-controlled digital games (1/10, 10%), and a virtual assistant–based CR program (1/10, 10%). A total of 40% (4/10) of the included studies [30-33] originated from the same RCT, the HF-Wii study. These publications represented secondary or subgroup analyses conducted in multinational cohorts (Sweden, Italy, the Netherlands, Israel, Germany, and the United States) that explored distinct outcome domains such as exercise capacity, psychological well-being, and cognitive predictors. To prevent double counting of data, these HF-Wii–derived studies were narratively synthesized and summarized separately rather than aggregated in quantitative comparisons.

### Risk of Bias

**Figure 2.**
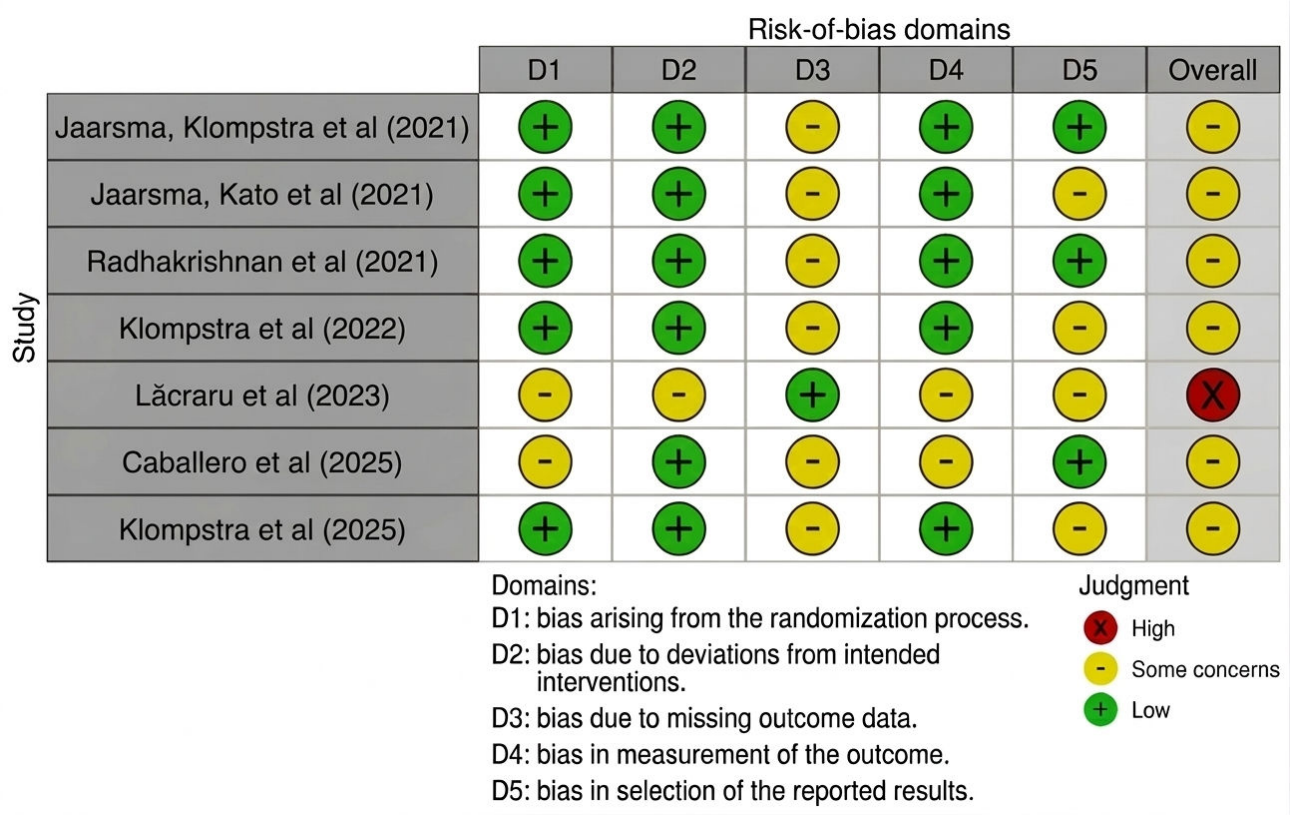
Summary of the risk-of-bias assessment using version 2 of the Cochrane risk-of-bias tool for randomized controlled trials.

**Figure 3.**
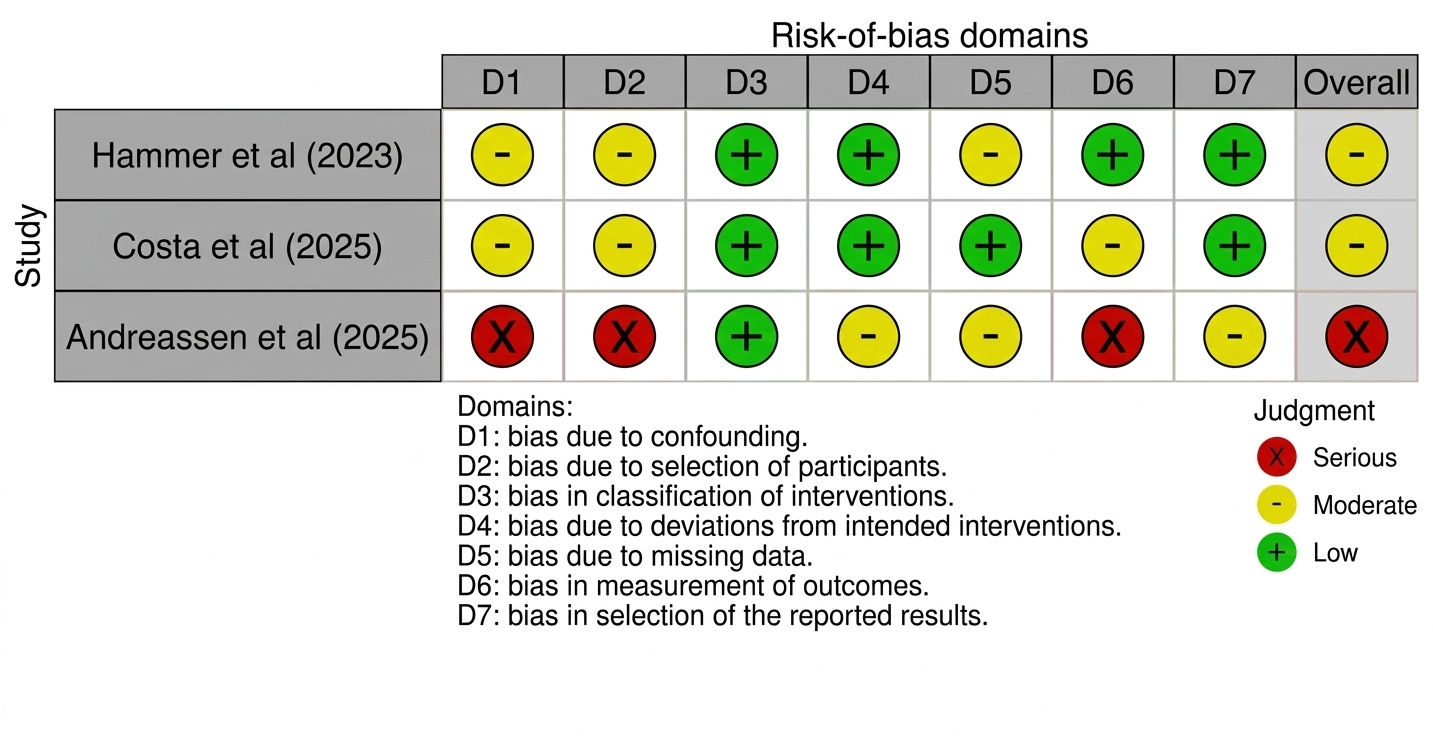
Summary of the risk-of-bias assessment using the Risk of Bias in Nonrandomized Studies of Interventions tool.

Of the 7 RCTs, 6 studies were rated as having some concerns, whereas 1 study showed a high risk of bias. Of the 3 non-RCTs, 2 studies showed a moderate risk of bias, whereas 1 feasibility study was judged to have a serious risk of bias. The summarized assessments are presented in Figure 2 [29-35] and in Figure 3 [26-28].

### Effects of VR-Based Interventions in Patients With HF

Table 2 and Multimedia Appendix 2 [26-35] show the detailed measurements, tools, and main results related to the effects of VR-based interventions in patients with HF.

**Table 2.** Outcome measurements of the included studies (N=10).

Variable^a^ and category	Studies, n (%)
Primary outcomes (physical activity related)	
Exercise capacity (6MWT^b^ and VO_2_ max^c^)	6 (60)
Exercise motivation	3 (30)
Exercise self-efficacy	3 (30)
Self-reported physical activity	2 (20)
Physical activity behaviors	2 (20)
Muscle strength	1 (10)
Daily walking distance	1 (10)
Mobilization experience	1 (10)
Sedentary time	1 (10)
Secondary outcomes	
Anxiety and depression	5 (50)
Quality of life	4 (40)
Well-being	2 (20)
HF^d^ self-management behaviors	2 (20)
HF self-management knowledge	1 (10)
HF self-efficacy	1 (10)
Cognitive function	1 (10)
HF hospitalization	1 (10)
Feasibility (acceptability, adherence, and retention)	3 (30)
Usability (user experience, system usability, and technology acceptance)	3 (30)
Safety	1 (10)

a In some cases, multiple reports or publications were derived from the same study. These entries share the same study population or dataset.

b 6MWT: 6-minute walk test.

c VO_2 _max: maximal oxygen consumption.

d HF: heart failure.

### Primary and Secondary Outcome Measurements

The most frequently assessed primary outcomes were exercise capacity—defined as physiological performance indicators such as the 6-minute walk test (6MWT) or maximal oxygen consumption (VO_2_ max)—reported in 60% (6/10) of the studies. In contrast, physical activity, treated as a distinct behavioral domain, was measured through self-reported activity levels, behavioral activity logs, muscle strength tests, mobilization experience, or sedentary time. Other primary outcomes included exercise motivation and self-efficacy (3/10, 30% of the studies each).

Secondary outcomes included anxiety and depression (5/10, 50%), QOL (4/10, 40%), well-being (2/10, 20%), and HF self-management behaviors (2/10, 20%), as well as intervention-related variables such as feasibility, usability, and safety.

### Effects on Physical Outcomes

Overall, VR-based interventions demonstrated positive trends in improving physical activity and exercise performance among patients with HF. In total, 50% (5/10) of the studies, which used home-based exergaming Nintendo Wii programs, reported improvements in 6MWT distances, with 2 of these studies showing statistically significant gains over 3 to 6 months (*P*<.05). For example, Klompstra et al [32] reported significant increases in exercise capacity and reductions in fatigue and dyspnea after 3 to 6 months, although these effects diminished by 12 months. Similarly, Hammer et al [26] found that VR exergaming improved 6MWT performance and QOL in patients with HF supported by left ventricular assist devices (*P*=.02). In addition, the mobile exergame (Heart Farming) demonstrated that daily walking targets were manageable and achievable, as confirmed by in-app tracking of walking distance, supporting the feasibility of integrating regular walking activity into daily routines [27].

The vCare virtual assistant app trial by Lăcraru et al [34] demonstrated significant improvements in VO_2_ max (*P*=.002) and reductions in low-density lipoprotein cholesterol (*P*<.05) compared with standard care.

Among hospitalized patients, Caballero et al [35] and Costa et al [28] found that immersive VR-assisted mobilization and exercise were safe, feasible, and associated with high levels of exercise enjoyment and positive usability ratings.

### Effects on Psychological and Cognitive Outcomes

Across the included studies, VR-based programs showed mixed but generally positive psychological effects. In total, 50% (5/10) of the studies assessed anxiety and depression, with 3 of these studies reporting significant postintervention reductions (*P*<.05). Lăcraru et al [34] found significant improvements in depression scores (*P*=.03), whereas anxiety levels remained unchanged. In contrast, Klompstra et al [32] observed no significant changes in anxiety or depression despite improvements in physical outcomes.

A total of 20% (2/10) of the studies evaluated overall well-being, reporting small to moderate improvements immediately following the intervention. Cognitive function was assessed in one secondary analysis [31], which identified lower baseline cognitive scores as a predictor of nonimprovement in exercise performance (odds ratio 0.87, 95% CI 0.80-0.94), suggesting that cognitive status may influence the effectiveness of VR-based interventions.

### Effects on Self-Management and QOL Outcomes

In total, 20% (2/10) of the studies focused on self-management behaviors. The sensor-controlled digital game intervention (Heart Health Mountain) by Radhakrishnan et al [29] significantly improved HF self-management knowledge (*P*<.05), QOL (*P*<.01), and adherence behaviors such as daily weight monitoring and physical activity (*r*=0.9; *P*<.001). Self-efficacy improved over 24 weeks, although between-group differences were not statistically significant. Additionally, reduced hospitalization rates were observed in both intervention and control groups over the 6-month follow-up period. The vCare digital coaching program [34] did not directly measure self-management behaviors but functioned as a self-management support tool. The intervention improved VO_2_ max (*P*=.002), QOL (*P*=.007), and depressive symptoms, whereas participants reported high usability and motivation for continued engagement in home-based rehabilitation.

QOL was assessed in 40% (4/10) of the studies [26,29,32,34], all of which reported improvements following VR-based interventions.

The Heart Health Mountain program [29] demonstrated significant increases in QOL at 6, 12, and 24 weeks (*P*<.01), along with enhanced self-efficacy and adherence to daily self-care activities.

Similarly, the vCare virtual assistant app [34] improved QOL (*P*=.007) and reduced depressive symptoms (*P*=.03), suggesting that personalized, feedback-driven home rehabilitation can yield psychosocial benefits in addition to physiological gains. In a study of left ventricular assist device–supported patients with HF, Hammer et al [26] found significant improvements in both exercise capacity and QOL after a 4-week home-based exergaming program.

Finally, Klompstra et al [32] observed notable improvements in QOL and reductions in fatigue and dyspnea during the 3- to 6-month follow-up period, although these effects diminished by 12 months.

### Feasibility, Usability, and Safety of VR-Based Interventions

Feasibility was reported in 30% (3/10) of the studies [26,27,29], showing high adherence, acceptability, and retention rates for home-based exergaming programs. Usability outcomes, assessed in the vCare [34] and VR-assisted cycling studies [28], indicated good to excellent user acceptance, with mean System Usability Scale scores exceeding 68 points. Similarly, the mobile exergame (Heart Farming) was perceived as easy to use, adaptable to individual needs, and engaging, with no major acceptability concerns reported [27]. Among hospitalized patients, immersive VR-assisted mobilization and exercise were also reported to be safe, feasible, and well tolerated [28,35].

No adverse events, cybersickness, or device-related safety concerns were reported in any of the included studies, indicating that VR-based interventions were well tolerated and safe for patients with HF.

## Discussion

### Principal Findings

This systematic review synthesized current evidence on the effects of VR-based interventions on physical activity, psychological outcomes, and self-management behaviors in patients with HF. Overall, the findings suggest that VR-based interventions can effectively enhance physical activity, exercise performance, and selected psychosocial outcomes in this population. Improvements in exercise capacity and QOL were the most consistently reported outcomes, while effects on psychological and behavioral domains were more variable across studies. The results also confirm the feasibility, usability, and safety of VR-based interventions in both hospital and home settings, demonstrating their potential as complementary or alternative strategies to traditional CR programs for patients with HF.

Consistent with prior meta-analyses on VR-based rehabilitation in chronic disease populations [20,36,37], the included studies demonstrated that VR can improve exercise capacity and physical activity levels in patients with HF. Exercise capacity, measured primarily through the 6MWT and VO_2_ max, showed significant improvements in several studies (4/10, 40%), indicating the physiological benefits of engaging in VR-mediated physical activity. Home-based exergaming programs, such as the HF-Wii intervention, encouraged patients to exercise safely and independently while maintaining adherence levels comparable to those of center-based CR [32]. These findings align with those of previous research reporting that gamified exercise enhances motivation, perceived enjoyment, and long-term engagement with physical activity among individuals with chronic cardiovascular conditions [6,21].

The ability of VR to provide immersive and interactive exercise experiences may help overcome common barriers to physical activity in HF populations, including fear of symptom exacerbation, low motivation, and limited access to supervised programs [7,8]. By integrating features such as real-time feedback, adjustable difficulty levels, and visual immersion, VR facilitates safe, individualized exercise experiences that enhance self-efficacy and engagement. Several trials (3/10, 30%) in this review reported high adherence rates (>85%) and positive user feedback regarding enjoyment, indicating that VR-based training is both acceptable and feasible for older adults. These results suggest that VR can serve as a practical extension of center-based CR carried out in the home, especially for individuals who face logistical or psychosocial barriers that hinder participation in traditional center-based programs.

Notably, the interventions in this review ranged from nonimmersive exergaming (eg, Nintendo Wii) to more immersive VR-assisted cycling systems. Although both formats generally demonstrated beneficial effects, the current evidence does not clearly indicate that higher immersion consistently produces superior outcomes, partly because intervention content, exercise intensity, and patient characteristics varied considerably across studies. Therefore, the level of immersion should be viewed as one of several interacting design features rather than the sole driver of effectiveness. Therefore, future research should explicitly compare different levels of immersion to identify which VR design characteristics—such as sensory stimulation, interaction fidelity, or gamification—are most strongly associated with adherence and clinical outcomes.

Nevertheless, the magnitude of improvement in exercise capacity varied across studies, and the sustainability of these effects beyond short-term follow-up remains uncertain. Some trials demonstrated attenuation of benefits after 6 to 12 months [32], implying that continued engagement or periodic reinforcement may be necessary to maintain gains. Future studies should explore strategies such as adaptive difficulty adjustment, remote coaching, and integration with wearable monitoring technologies to support long-term adherence and sustained physical activity.

In addition to physical outcomes, several studies (4/10, 40%) revealed potential psychological and cognitive benefits of VR-based interventions. Improvements in depressive symptoms and emotional well-being were observed in 30% (3/10) of the studies [26,29,34], consistent with previous findings showing that VR-based exercise can enhance mood, reduce stress, and foster social connectedness [11,17,38]. These effects may be mediated by mechanisms such as endorphin release from physical activity, distraction from illness-related distress, and enhanced self-efficacy through interactive goal attainment [39].

Moreover, VR environments provide an engaging, multisensory experience that may alleviate anxiety and fear associated with exercise, particularly among patients with HF who often perceive exertion as a potential trigger for worsening symptoms [4,5]. By enabling safe, controlled participation in virtual exercise, VR interventions may help reduce psychological resistance to activity. Therefore, the findings of this review support the potential of VR in promoting not only physical rehabilitation but also emotional adjustment and self-confidence in managing chronic illness.

Cognitive function, though less frequently assessed, appears to influence the efficacy of VR interventions. Jaarsma et al [30] reported that patients with lower baseline cognitive performance were less likely to achieve improvements in physical outcomes, suggesting that cognitive impairment may hinder engagement with VR technologies. This is likely because VR requires users to attend to visual-spatial cues, remember instructions, and coordinate motor actions; therefore, attention, working memory, and executive functioning play an important role in task performance and learning using VR [40]. When these abilities are reduced, patients may struggle to follow VR tasks or maintain engagement, which may attenuate treatment effects. Prior evidence indicates that cognitive decline, common in HF due to cerebral hypoperfusion, negatively affects self-care and rehabilitation adherence [41]. Accordingly, future research should consider cognitive screening to guide intervention tailoring and the potential value of incorporating cognitive training features into VR programs.

Although relatively few studies (2/10, 20%) examined self-management outcomes, there is available evidence suggesting that VR-based interventions can positively influence health-related behaviors and disease knowledge. The Heart Health Mountain program [29] demonstrated significant improvements in HF knowledge, QOL, and adherence to daily self-care tasks such as weight monitoring and physical activity logging. These results highlight VR’s educational potential—its interactive and gamified format can promote active learning, repetition, and reinforcement of self-management principles [15]. Integrating personalized behavioral feedback into VR platforms may further enhance self-regulation and motivation, supporting long-term lifestyle modification.

Nevertheless, behavior changes were inconsistent across studies. Some trials 2/10 (20%) reported only transient improvements that diminished over time, possibly due to limited intervention duration or insufficient strategies to maintain engagement [32]. Future research should investigate hybrid models that combine VR-based education with telemonitoring, remote coaching, or community support to promote sustained behavior change and long-term adherence. In addition, only 10% (1/10) of the studies used a clear theoretical framework to guide intervention design. Given that many VR programs aimed not only to increase physical activity but also to influence psychosocial or self-management behaviors, theory-driven approaches will be essential in future research to clarify mechanisms of change and enhance the sustainability of intervention effects.

One of the most encouraging findings of this review is the strong evidence for the feasibility, usability, and safety of VR-based interventions for patients with HF. No adverse cardiovascular events, cybersickness, or equipment-related injuries were reported across the 10 included studies. Adherence and retention rates were high, particularly in home-based interventions, indicating good acceptability among older adults. Usability assessments consistently yielded satisfactory results, suggesting that the VR systems used were intuitive and well tolerated even among participants with limited experience using digital technologies [18].

These findings are consistent with those of previous systematic reviews of VR-based rehabilitation in other chronic disease populations, where safety, enjoyment, and user engagement were identified as key facilitators of program success [16,20]. The use of commercially available, low-cost gaming systems (eg, Wii) in several studies (5/10, 50%) further supports the scalability and cost-effectiveness of VR interventions for HF management. As portable VR systems become more widely available, their integration into community and home settings will be increasingly feasible, offering opportunities to expand access to rehabilitation for underserved and mobility-limited populations.

Although 40% (4/10) of the included publications were derived from the same HF-Wii RCT, the findings across these reports were not identical. This variation can largely be explained by differences in follow-up duration and outcome selection. The main HF-Wii trial evaluated overall changes in exercise capacity at 3 months and reported no significant improvement, whereas the subsequent substudies targeted specific subgroups; additional follow-up periods (eg, 6 and 12 months); or secondary outcomes such as QOL, fatigue, and physical activity patterns. Consequently, some beneficial effects—particularly those observed at 3 to 6 months—appeared only in certain subanalyses and did not consistently persist across all publications. These differences highlight that the HF-Wii findings should not be interpreted as repeated independent evidence but rather as multiple analytic perspectives derived from a single parent dataset.

The findings of this review have several important implications. Clinically, VR-based interventions may serve as valuable adjuncts to traditional CR programs by offering flexible, engaging means to enhance physical activity and self-management, particularly for patients unable to attend in-person sessions. Health professionals can leverage VR platforms for remote monitoring, individualized goal setting, and motivational reinforcement. Integration of VR systems with wearable sensors and telehealth platforms could enable real-time feedback and progress tracking, fostering a more personalized and continuous rehabilitation experience [6].

From a research perspective, future RCTs should use larger sample sizes, extended follow-up periods, and standardized outcome metrics to allow for cross-study comparison and meta-analyses. Multidomain interventions combining physical, cognitive, and psychosocial training within VR environments may yield synergistic effects on overall health and well-being. Additionally, cost-effectiveness analyses and qualitative studies on patient experiences will be essential to inform clinical implementation and policy decisions. As VR technology continues to evolve, ensuring accessibility, ease of use, and cultural adaptability will be key to maximizing its impact on HF care.

### Limitations

Despite this systematic review presenting promising results, several methodological limitations should be acknowledged. First, the number of high-quality RCTs was limited, and most studies (4/10, 40%) involved small sample sizes, reducing statistical power and generalizability. Second, there was substantial heterogeneity in intervention design, duration, intensity, and outcome measures, which precluded meta-analysis and limited the ability to draw direct comparisons across studies. The diversity of VR modalities—ranging from nonimmersive exergames to fully immersive environments—also complicates interpretation of specific active components responsible for the observed benefits. Third, follow-up periods were typically short (4‐12 weeks), making it difficult to determine whether the observed improvements translate into sustained lifestyle changes or long-term clinical outcomes such as reduced hospitalization or mortality. Fourth, only 10% (1/10) of the studies used a theoretical framework to guide intervention development and behavioral engagement. Future studies should integrate behavior change theories and standardized outcome measures to strengthen conceptual and methodological rigor. In addition, 40% (4/10) of the included studies originated from the same parent trial (HF-Wii), resulting in overlapping participant samples and repeated use of the same trial dataset. To minimize potential bias from this overlap, these studies were treated as secondary analyses of a single RCT and were narratively summarized rather than combined quantitatively. Nonetheless, because these publications draw on the same underlying sample, their collective contribution should be interpreted with caution as they do not represent independent evidence. Finally, the search period was restricted to the last 10 years to reflect contemporary VR technology and current CR practice. While this approach increases clinical relevance to modern practice, it may also have excluded earlier pioneering VR studies.

### Conclusions

This systematic review advances the research field by providing the first focused synthesis of recent VR-based interventions specifically designed to promote physical activity in patients with HF while concurrently evaluating psychosocial and self-management outcomes. This study critically assessed research methodologies, intervention characteristics, and clinical effectiveness within the HF-specific context. By clarifying both the demonstrated short-term benefits and the methodological limitations of existing studies, this review contributes a conceptual and empirical road map for the future development of standardized VR-supported rehabilitation in HF. Importantly, the findings highlight the real-world potential of VR as a scalable, safe, and engaging home-based strategy capable of addressing persistent barriers to traditional CR. As digital health infrastructure continues to expand, evidence-based VR interventions could become an integral component of comprehensive HF self-management and rehabilitation programs. Nevertheless, because only 10% (1/10) of the included studies explicitly incorporated a formal behavior change theory, future large-scale, rigorously designed RCTs should integrate theoretical frameworks to guide intervention development and evaluation. Theory-driven designs will be essential for enhancing methodological rigor and ensuring the long-term sustainability of physical, psychosocial, and self-management outcomes in VR-based HF rehabilitation.

## Supplementary material

10.2196/86567 Multimedia Appendix 1 Search strategies.

10.2196/86567 Multimedia Appendix 2 Detailed data extraction of each study.

10.2196/86567 Checklist 1 PRISMA checklist.

10.2196/86567 Checklist 2 PRISMA-S checklist.

10.2196/86567 Checklist 3 SWiM reporting items.

## Abbreviations

6MWT: 6-minute walk test
CR: cardiac rehabilitation
HF: heart failure
MeSH: Medical Subject Headings
PRISMA: Preferred Reporting Items for Systematic Reviews and Meta-Analyses
PRISMA-S: Preferred Reporting Items for Systematic Reviews and Meta-Analyses extension for reporting literature searches
QOL: quality of life
RCT: randomized controlled trial
SWiM: Synthesis Without Meta-Analysis
VO_2_ max: maximal oxygen consumption
VR: virtual reality

## References

[1] McDonagh TA, Metra M, Authors/Task Force Members (2024). 2023 focused update of the 2021 ESC guidelines for the diagnosis and treatment of acute and chronic heart failure: developed by the task force for the diagnosis and treatment of acute and chronic heart failure of the European Society of Cardiology (ESC) with the special contribution of the Heart Failure Association (HFA) of the ESC. Eur J Heart Fail.

[2] Savarese G, Lund LH (2017). Global public health burden of heart failure. Card Fail Rev.

[3] Zheng X, Zheng Y, Ma J (2019). Effect of exercise-based cardiac rehabilitation on anxiety and depression in patients with myocardial infarction: a systematic review and meta-analysis. Heart Lung.

[4] Al-Sutari MM, Abdalrahim MS (2024). Symptom burden and quality of life among patients with heart failure. SAGE Open Nurs.

[5] Freedland KE, Carney RM, Rich MW, Steinmeyer BC, Skala JA, Dávila-Román VG (2016). Depression and multiple rehospitalizations in patients with heart failure. Clin Cardiol.

[6] Molloy C, Long L, Mordi IR (2024). Exercise-based cardiac rehabilitation for adults with heart failure. Cochrane Database Syst Rev.

[7] Dalal HM, Doherty P, Taylor RS (2015). Cardiac rehabilitation. BMJ.

[8] Ades PA, Keteyian SJ, Wright JS (2017). Increasing cardiac rehabilitation participation from 20% to 70%: a road map from the Million Hearts cardiac rehabilitation collaborative. Mayo Clin Proc.

[9] Anderson L, Oldridge N, Thompson DR (2016). Exercise-based cardiac rehabilitation for coronary heart disease: Cochrane systematic review and meta-analysis. J Am Coll Cardiol.

[10] Laver KE, Lange B, George S (2025). Virtual reality for stroke rehabilitation. Cochrane Database Syst Rev.

[11] Maples-Keller JL, Bunnell BE, Kim SJ, Rothbaum BO (2017). The use of virtual reality technology in the treatment of anxiety and other psychiatric disorders. Harv Rev Psychiatry.

[12] Goudman L, Jansen J, Billot M (2022). Virtual reality applications in chronic pain management: systematic review and meta-analysis. JMIR Serious Games.

[13] Hajesmaeel Gohari S, Gozali E, Niakan Kalhori SR (2019). Virtual reality applications for chronic conditions management: a review. Med J Islam Repub Iran.

[14] Tabassum A, Ghaznavi I, Abd-Alrazaq A, Qadir J (2025). Exploring the application of AI and extended reality technologies in metaverse-driven mental health solutions: scoping review. J Med Internet Res.

[15] Triberti S, Repetto C, Riva G (2014). Psychological factors influencing the effectiveness of virtual reality-based analgesia: a systematic review. Cyberpsychol Behav Soc Netw.

[16] Cano Porras D, Siemonsma P, Inzelberg R, Zeilig G, Plotnik M (2018). Advantages of virtual reality in the rehabilitation of balance and gait: systematic review. Neurology.

[17] Kim KA, Ahn JA (2024). Effectiveness of immersive virtual reality simulation programs using head-mounted displays in promoting physical activity in older adults: a systematic review. Clin Simul Nurs.

[18] Rodríguez-Mansilla J, Bedmar-Vargas C, Garrido-Ardila EM (2023). Effects of virtual reality in the rehabilitation of Parkinson’s disease: a systematic review. J Clin Med.

[19] Matamala-Gomez M, Donegan T, Bottiroli S, Sandrini G, Sanchez-Vives MV, Tassorelli C (2019). Immersive virtual reality and virtual embodiment for pain relief. Front Hum Neurosci.

[20] Rutkowski S, Rutkowska A, Kiper P (2020). Virtual reality rehabilitation in patients with chronic obstructive pulmonary disease: a randomized controlled trial. Int J Chron Obstruct Pulmon Dis.

[21] García-Bravo S, Cuesta-Gómez A, Campuzano-Ruiz R (2021). Virtual reality and video games in cardiac rehabilitation programs. A systematic review. Disabil Rehabil.

[22] Page MJ, McKenzie JE, Bossuyt PM (2021). The PRISMA 2020 statement: an updated guideline for reporting systematic reviews. J Clin Epidemiol.

[23] Rethlefsen ML, Kirtley S, Waffenschmidt S (2021). PRISMA-S: an extension to the PRISMA statement for reporting literature searches in systematic reviews. Syst Rev.

[24] Sterne JAC, Savović J, Page MJ (2019). RoB 2: a revised tool for assessing risk of bias in randomised trials. BMJ.

[25] Sterne JA, Hernán MA, Reeves BC (2016). ROBINS-I: a tool for assessing risk of bias in non-randomised studies of interventions. BMJ.

[26] Hammer Y, Shaul AA, Ben-Avraham B (2023). Exergaming in patients with a left ventricular assist device: a feasibility study. ESC Heart Fail.

[27] Andreassen M, Santaularia N, Berglund A (2025). Feasibility of a mobile exergame for patients with heart failure. Games Health J.

[28] Costa AS, Barbosa CB, Guizilini S (2025). Virtual reality and physical activity in patients with heart failure: technology validation and user satisfaction – pilot study. Int J Cardiovasc Sci.

[29] Radhakrishnan K, Julien C, Baranowski T (2021). Feasibility of a sensor-controlled digital game for heart failure self-management: randomized controlled trial. JMIR Serious Games.

[30] Jaarsma T, Klompstra L, Ben Gal T (2021). Effects of exergaming on exercise capacity in patients with heart failure: results of an international multicentre randomized controlled trial. Eur J Heart Fail.

[31] Jaarsma T, Perkiö Kato N, Ben Gal T (2021). Factors associated with lack of improvement in submaximal exercise capacity of patients with heart failure. ESC Heart Fail.

[32] Klompstra L, Hägglund E, Jaarsma T, Kato NP, Strömberg A (2025). Effects of exergaming and yoga on exercise capacity and physical and mental health in heart failure patients: a randomized sub-study. Eur J Cardiovasc Nurs.

[33] Klompstra L, Jaarsma T, Piepoli MF (2022). Objectively measured physical activity in patients with heart failure: a sub-analysis from the HF-Wii study. Eur J Cardiovasc Nurs.

[34] Lăcraru AE, Busnatu Ștefan S, Pană MA (2023). Assessing the efficacy of a virtual assistant in the remote cardiac rehabilitation of heart failure and ischemic heart disease patients: case-control study of Romanian adult patients. Int J Environ Res Public Health.

[35] Caballero LG, Fraga IB, Tremea CEM (2025). Experience of heart failure patients in mobilization with virtual reality: mixed methods study. Rev Esc Enferm USP.

[36] Cugusi L, Prosperini L, Mura G (2021). Exergaming for quality of life in persons living with chronic diseases: a systematic review and meta-analysis. PM R.

[37] Kwon SH, Park JK, Koh YH (2023). A systematic review and meta-analysis on the effect of virtual reality-based rehabilitation for people with Parkinson’s disease. J Neuroeng Rehabil.

[38] García-Betances RI, Jiménez-Mixco V, Arredondo MT, Cabrera-Umpiérrez MF (2015). Using virtual reality for cognitive training of the elderly. Am J Alzheimers Dis Other Demen.

[39] Martín-Rodríguez A, Gostian-Ropotin LA, Beltrán-Velasco AI (2024). Sporting mind: the interplay of physical activity and psychological health. Sports (Basel).

[40] Lachowicz M, Serweta-Pawlik A, Żurek A (2025). Application of short and long-term virtual reality training for visuospatial memory development in amateur e-athletes. Virtual Real.

[41] Goh FQ, Kong WKF, Wong RCC (2022). Cognitive impairment in heart failure-a review. Biology (Basel).

