# Supplementary material for: Effects of Virtual Reality–Based Interventions for Promoting Physical Activity in Patients With Heart Failure: Systematic Review
Source: J Med Internet Res. 2026 Mar 24;28:e86567. doi: 10.2196/86567 (PMC13012233; doi:10.2196/86567)
Supplement: Multimedia Appendix 1 [file jmir-v28-e86567-s001.pdf]

Supplement 1. Search Strategies

**1. PubMed**	
No.	Query
#6	#1 AND (#2 OR #3 OR #4) AND #5
#5	"Exercise"[mesh] OR "Exercise Therapy"[mesh] OR "exercis*"[tiab] OR "workout"[tiab] OR "work-out"[tiab] OR "physical activit*"[tiab] OR "physical training"[tiab] OR "fitness"[tiab] OR "motion track*"[tiab] OR ("physical*"[ti] AND ("activit*"[ti] OR "workout"[ti] OR "work-out"[ti] OR "training"[ti])) OR "kinesiotherapy*"[tiab] OR "behavio* chang*"[tiab] OR "self care behavio*"[tiab] OR "selfcare behavio*"[tiab] OR "self caring behavio*"[tiab] OR "self manag* behavio*"[tiab] OR "health* behavio*"[tiab] OR "health care* behavio*"[tiab] OR (("self care*"[ti] OR "selfcare*"[ti] OR "self caring*"[ti] OR "selfcaring*"[ti] OR "self manag*"[ti] OR "selfmanag*"[ti] OR "health*"[ti] OR "health care*"[ti]) AND "behavio*"[ti]) OR "selfcare behavior"[tiab:~3] OR "selfcare behaviors"[tiab:~3] OR "selfcare behaviour"[tiab:~3] OR "selfcare behaviours"[tiab:~3] OR "self care behavior"[tiab:~3] OR "self care behaviors"[tiab:~3] OR "self care behaviour"[tiab:~3] OR "self care behaviours"[tiab:~3] OR "self management behavior"[tiab:~3] OR "self management behaviors"[tiab:~3] OR "self management behaviour"[tiab:~3] OR "self management behaviours"[tiab:~3] OR "health behavior"[tiab:~3] OR "health behaviors"[tiab:~3] OR "health behaviour"[tiab:~3] OR "health behaviours"[tiab:~3] OR "healthcare behavior"[tiab:~3] OR "healthcare behaviors"[tiab:~3] OR "healthcare behaviour"[tiab:~3] OR "healthcare behaviours"[tiab:~3] OR "health care behavior"[tiab:~3] OR "health care behaviors"[tiab:~3] OR "health care behaviour"[tiab:~3] OR "health care behaviours"[tiab:~3]
#4	"Smart Glasses"[mesh] OR "smart glass*"[tiab] OR "smartglass*"[tiab] OR "head mount* display*"[tiab] OR ("HMD"[tiab] AND "display*"[tiab]) OR "head up display*"[tiab] OR "head worn display*"[tiab] OR "google glass*"[tiab] OR (("Wii"[tiab] OR "Xbox"[tiab] OR "Kinect"[tiab] OR "Playstation"[tiab] OR "Vive"[tiab]) AND ("Video Games"[mesh] OR "video game*"[tiab] OR "videogame*"[tiab] OR "video gaming*"[tiab] OR "videogaming*"[tiab] OR "Nintendo"[tiab] OR "Microsoft"[tiab] OR "Sony"[tiab] OR "HTC"[tiab])) OR "Nintendo"[tiab] OR "Apple Vision"[tiab] OR "Meta Quest"[tiab] OR "Oculus"[tiab] OR (("Video Games"[mesh] OR "game"[ti] OR "games"[ti] OR "gaming*"[ti]) AND ("virtual*"[tiab] OR "avatar*"[tiab] OR "three-dimension*"[tiab] OR "3-dimension*"[tiab] OR "3D"[tiab] OR "3Ds"[tiab]))
#3	"Exergaming"[mesh] OR "exergame*"[tiab] OR "exergaming*"[tiab] OR "exer-game*"[tiab] OR "exer-gaming*"[tiab] OR "virtual reality game*"[tiab] OR "virtual reality gaming*"[tiab] OR "virtual reality exercis*"[tiab] OR "virtual reality-based exercis*"[tiab] OR "augmented reality game*"[tiab] OR "mixed reality game*"[tiab] OR "VR game*"[tiab] OR "VR gaming*"[tiab] OR "VR exergame*"[tiab] OR "VR exergaming*"[tiab] OR "VR exercis*"[tiab] OR "VR-based game*"[tiab] OR "VR-based exergaming*"[tiab] OR "VR-based exercis*"[tiab] OR "AR game*"[tiab] OR "AR-based exercis*"[tiab] OR "active-video game*"[tiab] OR "active-video gaming*"[tiab] OR "active videogame*"[tiab] OR "active videogaming*"[tiab]

#2	"Virtual Reality"[mesh] OR "Virtual Reality Exposure Therapy"[mesh] OR "Augmented Reality"[mesh] OR "virtual realit*"[tiab] OR "avatar*"[tiab] OR "augmented realit*"[tiab] OR "mixed realit*"[tiab] OR (("VR"[tiab] OR "AR"[tiab] OR "MR"[tiab] OR "XR"[tiab]) AND ("virtual*"[tiab] OR "augment*"[tiab] OR "mixed"[tiab] OR "extend*"[tiab]) AND "realit*"[tiab])
#1	"Heart Failure"[mesh] OR "heart failure"[tiab] OR "heart decompensation"[tiab] OR "heart insufficienc*"[tiab] OR "cardi* failure"[tiab] OR "cardi* decompensation"[tiab] OR "cardi* incompetenc*"[tiab] OR "cardi* insufficienc*"[tiab] OR "myocardi* failure"[tiab] OR "myocardi* decompensation"[tiab] OR "myocardi* incompetenc*"[tiab] OR "myocardi* insufficienc*"[tiab] OR (("heart"[ti] OR "cardiac*"[ti] OR "cardio*"[ti] OR "cardial"[ti] OR "myocardi*"[ti]) AND ("failure"[ti] OR "decompensation"[ti] OR "incompetenc*"[ti] OR "insufficienc*"[ti]))
**2. Embase**	
No.	Query
#6	#1 AND (#2 OR #3 OR #4) AND #5
#5	('exercise'/exp OR 'kinesiotherapy'/exp OR 'exercis*':ti,ab,kw OR 'workout':ti,ab,kw OR 'work-out':ti,ab,kw OR 'physical activit*':ti,ab,kw OR 'physical training':ti,ab,kw OR 'fitness':ti,ab,kw OR 'motion track*':ti,ab,kw OR ('physical*':ti,kw AND ('activit*':ti,kw OR 'workout':ti,kw OR 'work-out':ti,kw OR 'training':ti,kw)) OR 'kinesiotherapy*':ti,ab,kw OR 'behavio* chang*':ti,ab,kw OR ((('self care*' OR 'selfcare*' OR 'self caring*' OR 'selfcaring*' OR 'self manag*' OR 'selfmanag*' OR 'health*' OR 'health care*') NEAR/3 'behavio*'):ti,ab,kw))
#4	('virtual reality head mounted display'/exp OR 'smart glasses'/exp OR 'smart glass*':ti,ab,kw OR 'smartglass*':ti,ab,kw OR 'head mount* display*':ti,ab,kw OR ('hmd':ti,ab,kw AND 'display*':ti,ab,kw) OR 'head up display*':ti,ab,kw OR 'head worn display*':ti,ab,kw OR 'google glass*':ti,ab,kw OR (('wii':ti,ab,kw OR 'xbox':ti,ab,kw OR 'kinect':ti,ab,kw OR 'playstation':ti,ab,kw OR 'vive':ti,ab,kw) AND ('video game'/exp OR 'video game*':ti,ab,kw OR 'videogame*':ti,ab,kw OR 'video gaming*':ti,ab,kw OR 'videogaming*':ti,ab,kw OR 'nintendo':ti,ab,kw OR 'microsoft':ti,ab,kw OR 'sony':ti,ab,kw OR 'htc':ti,ab,kw)) OR 'nintendo':ti,ab,kw OR 'apple vision':ti,ab,kw OR 'meta quest':ti,ab,kw OR 'oculus':ti,ab,kw OR (('video game'/exp OR 'game':ti,kw OR 'games':ti,kw OR 'gaming*':ti,kw) AND ('virtual*':ti,ab,kw OR 'avatar*':ti,ab,kw OR 'three-dimension*':ti,ab,kw OR '3-dimension*':ti,ab,kw OR '3d':ti,ab,kw OR '3ds':ti,ab,kw)))
#3	('exergaming'/exp OR 'exergame'/exp OR 'exergame*':ti,ab,kw OR 'exergaming*':ti,ab,kw OR 'exer-game*':ti,ab,kw OR 'exer-gaming*':ti,ab,kw OR 'active-video game*':ti,ab,kw OR 'active-video gaming*':ti,ab,kw OR 'active videogame*':ti,ab,kw OR 'active videogaming*':ti,ab,kw OR (((('virtual realit*' OR 'vr' OR 'augmented realit*' OR 'ar' OR 'mixed realit*' OR 'mr' OR 'extended realit*' OR 'xr') NEXT/2 ('game*' OR 'gaming*' OR 'exergame*' OR 'exergaming*' OR 'exercis*' OR 'based game*' OR 'based gaming*' OR 'based exergame*' OR 'based exergaming*' OR 'based exercis*')):ti,ab,kw) AND 'realit*':ti,ab,kw))

#2	('virtual reality'/exp OR 'virtual reality system'/exp OR 'virtual reality exposure therapy'/exp OR 'augmented reality'/exp OR 'augmented reality system'/exp OR 'mixed reality'/exp OR 'mixed reality technology'/exp OR 'virtual realit*':ti,ab,kw OR 'avatar*':ti,ab,kw OR 'augmented realit*':ti,ab,kw OR 'mixed realit*':ti,ab,kw OR (('vr':ti,ab,kw OR 'ar':ti,ab,kw OR 'mr':ti,ab,kw OR 'xr':ti,ab,kw) AND ('virtual*':ti,ab,kw OR 'augment*':ti,ab,kw OR 'mixed':ti,ab,kw OR 'extend*':ti,ab,kw) AND 'realit*':ti,ab,kw))
#1	('heart failure'/exp OR ((('heart' OR 'cardiac*' OR 'cardio*' OR 'cardial' OR 'myocardi*') NEAR/3 ('failure' OR 'decompensat*' OR 'incompetenc*' OR 'insufficienc*')):ti,ab,kw) OR (('heart':ti,kw OR 'cardiac*':ti,kw OR 'cardio*':ti,kw OR 'cardial':ti,kw OR 'myocardi*':ti,kw) AND ('failure':ti,kw OR 'decompensat*':ti,kw OR 'incompetenc*':ti,kw OR 'insufficienc*':ti,kw)))
**3. CINAHL**	
No.	Query
S6	S1 AND (S2 OR S3 OR S4) AND S5
S5	(MH Exercise+) OR (MH Therapeutic Exercise+) OR TI ("exercis*" OR "workout" OR "work-out" OR "physical activit*" OR "physical training" OR "fitness" OR "motion track*" OR ("physical*" AND ("activit*" OR "workout" OR "work-out" OR "training")) OR "kinesiotherapy*" OR "behavio* chang*" OR (("self care*" OR "selfcare*" OR "self caring*" OR "selfcaring*" OR "self manag*" OR "selfmanag*" OR "health*" OR "health care*") N3 "behavio*")) OR AB ("exercis*" OR "workout" OR "work-out" OR "physical activit*" OR "physical training" OR "fitness" OR "motion track*" OR "kinesiotherapy*" OR "behavio* chang*" OR (("self care*" OR "selfcare*" OR "self caring*" OR "selfcaring*" OR "self manag*" OR "selfmanag*" OR "health*" OR "health care*") N3 "behavio*"))
S4	(MH Smart Glasses+) OR TI ("smart glass*" OR "smartglass*" OR "head mount* display*" OR ("HMD" AND "display*") OR "head up display*" OR "head worn display*" OR "google glass*") OR AB ("smart glass*" OR "smartglass*" OR "head mount* display*" OR ("HMD" AND "display*") OR "head up display*" OR "head worn display*" OR "google glass*") OR ((TI ("Wii" OR "Xbox" OR "Kinect" OR "Playstation" OR "Vive") OR AB ("Wii" OR "Xbox" OR "Kinect" OR "Playstation" OR "Vive")) AND ((MH Video Games+) OR TI ("video game*" OR "videogame*" OR "video gaming*" OR "videogaming*" OR "Nintendo" OR "Microsoft" OR "Sony" OR "HTC") OR AB ("video game*" OR "videogame*" OR "video gaming*" OR "videogaming*" OR "Nintendo" OR "Microsoft" OR "Sony" OR "HTC"))) OR TI ("Nintendo" OR "Apple Vision" OR "Meta Quest" OR "Oculus") OR AB ("Nintendo" OR "Apple Vision" OR "Meta Quest" OR "Oculus") OR (((MH Video Games+) OR TI ("game" OR "games" OR "gaming*")) AND (TI ("virtual*" OR "avatar*" OR "three-dimension*" OR "3-dimension*" OR "3D" OR "3Ds") OR AB ("virtual*" OR "avatar*" OR "three-dimension*" OR "3-dimension*" OR "3D" OR "3Ds")))
S3	(MH Exergames+) OR TI ("exergame*" OR "exergaming*" OR "exer-game*" OR "exer-gaming*" OR "active-video game*" OR "active-video gaming*"

	OR "active videogame*" OR "active videogaming*" OR ((("virtual realit*" OR "VR" OR "augmented realit*" OR "AR" OR "mixed realit*" OR "MR" OR "extended realit*" OR "XR") W2 ("game*" OR "gaming*" OR "exergame*" OR "exergaming*" OR "exercis*" OR "based game*" OR "based gaming*" OR "based exergame*" OR "based exergaming*" OR "based exercis*")) AND "realit*")) OR AB ("exergame*" OR "exergaming*" OR "exer-game*" OR "exer-gaming*" OR "active-video game*" OR "active-video gaming*" OR "active videogame*" OR "active videogaming*" OR ((("virtual realit*" OR "VR" OR "augmented realit*" OR "AR" OR "mixed realit*" OR "MR" OR "extended realit*" OR "XR") W2 ("game*" OR "gaming*" OR "exergame*" OR "exergaming*" OR "exercis*" OR "based game*" OR "based gaming*" OR "based exergame*" OR "based exergaming*" OR "based exercis*")) AND "realit*"))
S2	(MH Virtual Reality+) OR (MH Avatars+) OR (MH Virtual Reality Exposure Therapy+) OR (MH Augmented Reality+) OR TI ("virtual realit*" OR "avatar*" OR "augmented realit*" OR "mixed realit*" OR (("VR" OR "AR" OR "MR" OR "XR") AND ("virtual*" OR "augment*" OR "mixed" OR "extend*") AND "realit*")) OR AB ("virtual realit*" OR "avatar*" OR "augmented realit*" OR "mixed realit*" OR (("VR" OR "AR" OR "MR" OR "XR") AND ("virtual*" OR "augment*" OR "mixed" OR "extend*") AND "realit*"))
S1	(MH Heart Failure+) OR TI (("heart" OR "cardiac*" OR "cardio*" OR "cardial" OR "myocardi*") AND ("failure" OR "decompensat*" OR "incompetenc*" OR "insufficienc*")) OR AB (("heart" OR "cardiac*" OR "cardio*" OR "cardial" OR "myocardi*") N3 ("failure" OR "decompensat*" OR "incompetenc*" OR "insufficienc*"))
**4. Scopus**	
No.	Query
6	#1 AND (#2 OR #3 OR #4) AND #5
5	KEY ("Exercise" OR "Exercise Therapy" OR "Kinesiotherapy" OR "Therapeutic Exercise") OR TITLE-ABS-KEY ("exercis*" OR "workout" OR "work-out" OR "physical activit*" OR "physical training" OR "fitness" OR "motion track*" OR "kinesiotherapy*" OR "behavio* chang*" OR (("self care*" OR "selfcare*" OR "self caring*" OR "selfcaring*" OR "self manag*" OR "selfmanag*" OR "health*" OR "health care*") W/3 "behavio*")) OR ((TITLE ("physical*") OR KEY ("physical*")) AND (TITLE ("activit*" OR "workout" OR "work-out" OR "training") OR KEY ("activit*" OR "workout" OR "work-out" OR "training")))
4	KEY ("Smart Glasses" OR "Virtual Reality Head Mounted Display") OR TITLE-ABS-KEY ("smart glass*" OR "smartglass*" OR "head mount* display*" OR ("HMD" AND "display*") OR "head up display*" OR "head worn display*" OR "google glass*") OR (TITLE-ABS-KEY ("Wii" OR "Xbox" OR "Kinect" OR "Playstation" OR "Vive") AND (KEY ("Video Games" OR "Video Game") OR TITLE-ABS-KEY ("video game*" OR "videogame*" OR "video gaming*" OR "videogaming*" OR "Nintendo" OR "Microsoft" OR "Sony" OR "HTC"))) OR TITLE-ABS-KEY ("Nintendo"

	OR "Apple Vision" OR "Meta Quest" OR "Oculus") OR ((KEY ("Video Games" OR "Video Game") OR TITLE ("game" OR "games" OR "gaming*") OR KEY ("game" OR "games" OR "gaming*")) AND (TITLE-ABS-KEY ("virtual*" OR "avatar*" OR "three-dimension*" OR "3-dimension*" OR "3D" OR "3Ds")))
3	KEY ("Exergaming" OR "Exergame" OR "Exergames") OR TITLE-ABS-KEY ("exergame*" OR "exergaming*" OR "exer-game*" OR "exer-gaming*" OR "active-video game*" OR "active-video gaming*" OR "active videogame*" OR "active videogaming*" OR ((("virtual realit*" OR "VR" OR "augmented realit*" OR "AR" OR "mixed realit*" OR "MR" OR "extended realit*" OR "XR") PRE/2 ("game*" OR "gaming*" OR "exergame*" OR "exergaming*" OR "exercis*" OR "based game*" OR "based gaming*" OR "based exergame*" OR "based exergaming*" OR "based exercis*")) AND "realit*"))
2	KEY ("Virtual Reality" OR "Virtual Reality Exposure Therapy" OR "Augmented Reality" OR "Virtual Reality System" OR "Augmented Reality System" OR "Mixed Reality" OR "Mixed Reality Technology" OR "Avatars") OR TITLE-ABS-KEY ("virtual realit*" OR "avatar*" OR "augmented realit*" OR "mixed realit*" OR (("VR" OR "AR" OR "MR" OR "XR") AND ("virtual*" OR "augment*" OR "mixed" OR "extend*") AND "realit*"))
1	KEY ("Heart Failure") OR TITLE-ABS-KEY (("heart" OR "cardiac*" OR "cardio*" OR "cardial" OR "myocardi*") W/3 ("failure" OR "decompensat*" OR "incompetenc*" OR "insufficienc*")) OR ((TITLE ("heart" OR "cardiac*" OR "cardio*" OR "cardial" OR "myocardi*") OR KEY ("heart" OR "cardiac*" OR "cardio*" OR "cardial" OR "myocardi*")) AND (TITLE ("failure" OR "decompensat*" OR "incompetenc*" OR "insufficienc*") OR KEY ("failure" OR "decompensat*" OR "incompetenc*" OR "insufficienc*")))
