# Supplementary material for: Effects of Virtual Reality–Based Interventions for Promoting Physical Activity in Patients With Heart Failure: Systematic Review
Source: J Med Internet Res. 2026 Mar 24;28:e86567. doi: 10.2196/86567 (PMC13012233; doi:10.2196/86567)
Supplement: Multimedia Appendix 2 [file jmir-v28-e86567-s002.pdf]

Table1. Detailed Data Extraction of Each Study (N = 10)

**Author (year) / Country**	**Study aim**	**Research design**	**Theoretical framework**	**Population**				**Intervention detail**	**Measurements & tools**	**Follow-up**	**Main findings**
				**Sample size (EG/CG)**	**Drop-out (EG/CG)**	**Gender (M:F) (%)**	**Mean age**				
Jaarsma, Klompstra et al. (2021) / Multinational (Sweden, Italy, Netherlands, Israel, Germany, USA) [27]	To evaluate effects of home-based exergaming (HF-Wii) on exercise capacity, muscle function, self-reported PA, and exercise motivation in patients with HF	Multicenter RCT	No	605 (305/300)	82 withdrew; 3 deaths during trial	429:176 (71:29)	67±12	• EG: Home-based exergaming using Nintendo Wii, 30 min/day, 5 days/week, for 3 months, plus regular telephone support<br>• CG: Standard care and PA advice with follow-up calls	• Exercise capacity: 6MWT<br>• Muscle strength: isotonic shoulder flexion, isotonic heel-lift<br>• Exercise motivation: Exercise Motivation Index<br>• Exercise self-efficacy: Exercise Self-Efficacy questionnaire<br>• Self-reported PA: Single item question on activity of the last week	• Baseline<br>• Post 1 (3 months)<br>• Post 2 (6 months)<br>• Post 3 (1 year)	• 6MWT distance was slightly higher in EG at 3, 6, and 12 months, but differences were not statistically significant after baseline adjustment.<br>• A slight but statistically significant improvement in heel-raise muscle strength in EG.<br>• No significant differences in exercise motivation, self-efficacy, or self-reported PA.
Jaarsma, Kato et al. (2021) / Multinational (Sweden, Italy, Netherlands, Israel, Germany, USA) [28]	To identify factors associated with lack of improvement in submaximal exercise capacity of exergame (HF-Wii) in patients with HF	Secondary analysis of Multicenter RCT	No	480 (238/242)	125 excluded from original RCT (n=605)	346:134 (72:28)	67	• EG: Home-based exergaming using Nintendo Wii, 30 min/day, 5 days/week, for 3 months, plus regular telephone support<br>• CG: Standard care and PA advice with follow-up calls	• Exercise capacity: 6MWT<br>• Self-reported PA: Single item question on activity of the last week<br>• Cognitive function: Montreal Cognitive Assessment<br>• NYHA class<br>• Serum creatinine<br>• Exercise motivation: Exercise Motivation Index<br>• Exercise self-efficacy: Exercise Self-Efficacy questionnaire	• Baseline<br>• Post (3 months)	• Overall: 33% of participants improved ≥30 m in the 6MWT.<br>• Among those with a baseline distance ≥300 m, 18% deteriorated by >30 m.<br>• Predictors of non-improvement: Lower baseline PA (OR=0.77), lower cognitive function (OR=0.87).<br>• Among baseline <300 m group: NYHA III/IV (OR=4.68), higher serum creatinine (OR=1.02), lower cognitive function (OR=0.86), and lower anxiety (OR=0.84) predicted lack of improvement.

Radhakrishnan et al. (2021) / USA [26]	To evaluate efficacy of sensor-controlled digital game intervention (Heart Health Mountain) for improving self-management behaviors and knowledge in patients with HF	RCT	Yes (Fogg Behavior Model)	38 (19/19)	11 (5/6)	20:18 (53:47)	Not reported (participants ≥55 years, 47% were aged 65 years or older)	• EG: Sensor-controlled mobile digital game with real-time feedback via weight and activity sensors. The game encouraged adherence through tasks and rewards over 12 weeks.<br>• CG: Usual care including standard HF self-care education. Received the same sensors but no game.	• Weight monitoring behavior: collected from sensor logs within Health Mate app<br>• PA behavior: daily step count and active minutes measured by sensor logs within Health Mate app<br>• HF-related functional status: Items 1-12 of Kansas City Cardiomyopathy Questionnaire (KCCQ)<br>• QOL: Items 13-15 of KCCQ<br>• HF self-management knowledge: Atlanta Heart Failure Knowledge Test<br>• Self-reported HF self-care behaviors: European Heart Failure Self-care Behavior Scale<br>• HF self-efficacy: Subscale of Self-Care of Heart Failure Index<br>• Motivation for HF self-management behaviors: Treatment Self-Regulation Questionnaire<br>• HF hospitalization: Participants' self-report<br>• Feasibility metrics: Retention rate, Engagement and adherence rate<br>• Satisfaction: Subscale of Intrinsic	• Baseline<br>• Post 1 (6 weeks)<br>• Post 2 (3 months)<br>• Post 3 (6 months)	• The number of days each player opened the game app was strongly associated with the number of days the player engaged in weight monitoring (r=0.72; p=.04) and the number of days with PA step data (r=0.9; p<.001).<br>• EG showed significant improvement in self-reported weight monitoring, with the average number of days of weight monitoring being 46% higher than that of CG (p<.001).<br>• EG showed significant improvement in QOL at 6, 12, and 24 weeks (p<.01).<br>• EG also showed improvement in HF self-management knowledge, with statistically significant results at 6, 12, and 24 weeks (p<.05).<br>• EG showed significant improvement in HF self-efficacy at 6, 12, and 24 weeks, but CG showed a trend towards greater improvement (p<.05).<br>• The change in motivation for HF self-management behaviors was not statistically significant in either group.<br>• There was a reduction in 6-month hospitalization rates in both groups.<br>• Satisfaction survey reported that the sensor-controlled digital game was easy to use.

									Motivation Inventory		
Klompstra et al. (2022) / Multinational (Sweden, Netherlands, Germany, USA) [30]	To explore objectively measured PA and sedentary time in HF patients with exergaming (HF-Wii) and predictors of activity	Sub-analysis of Multicenter RCT	No	64 (28/36)	NA	47:17 (73:27)	69±9	• EG: Home-based exergaming using Nintendo Wii, 30 min/day, 5 days/week, for 3 months, plus regular telephone support<br>• CG (Motivational support group): Standard care and PA advice with follow-up calls	• Accelerometer for sedentary time and PA<br>• Exercise capacity: 6MWT<br>• Exercise self-efficacy: Exercise Self-Efficacy questionnaire<br>• Exercise motivation: Exercise Motivation Index<br>• Anxiety and depression: HADS	• Baseline<br>• Post (3 months)	• EG showed a decrease of 28 minutes on average after 3 months, while CG increased by 2 minutes. However, this change was not statistically significant.<br>• No significant differences in objectively measured PA or sedentary time between groups.<br>• Predictors of increased activity: Having grandchildren (OR=7.43), recent HF diagnosis (OR=0.93), and higher social motivation (OR=2.31).
Hammer et al. (2023) / Israel [31]	To evaluate exercise capacity, feasibility, and safety of exergaming in LVAD-supported HF patients	One group (single arm) trial	No	11	5	10:1 (91:9)	67±7	Home-based exergaming program (Nintendo Wii Sports console including bowling, tennis, golf, boxing, and baseball), 30 min/day, 5 days/week, for 4 weeks	• Exercise capacity: 6MWT<br>• QOL: Minnesota Living with Heart Failure Questionnaire (MLHFQ), Cantril's Ladder of Life<br>• Feasibility & safety: Patient self-reported diaries and semi-structured interview	• Baseline<br>• Post (1 month)	• EG showed significant improvements in 6MWT distance (p=.023)<br>• EG showed significant improvements in QOL.<br>• No specific safety issues related to LVAD have been reported, and patients most often engaged with and enjoyed the intervention.
Lăcraru et al. (2023) / Romania [32]	To evaluate effectiveness of virtual assistant app (vCare) in motivating	RCT	No	50 (HF: 30; IHD: 20) (HF [EG/A	3 (EG)	33:17 (66:34)	HF: 61.5±9.4; IHD: 58.1±7.1	• EG: Home-based rehabilitation guided by the vCare virtual assistant app, with	• Exercise capacity: VO_2max<br>• Clinical outcome: blood pressure, lipid profile<br>• QOL: MLHFQ<br>• Anxiety and	• Baseline<br>• Post (3 months)	<HF group><br>• EG showed significant improvements in exercise capacity (VO_2max) (p=.002); decreased in CG.<br>• LDL cholesterol significantly fell in EG; minimal change in CG.

	patients with HF or IHD to actively engage in personalized cardiac rehabilitation to improve independence and QOL			mbulatory rehab group/ CG=10 /10/10] ; IHD [EG/CG=10/10])				individualized programs including exercise, medication reminders, lifestyle education, psychological support, and risk-factor monitoring, daily, for 3 months<br>• Ambulatory rehab group: Conventional cardiac rehab at hospital<br>• CG: Received advice at discharge how to perform cardiac rehab at home	depression: HADS<br>• Physical addiction to nicotine : Fagerstrom Test<br>• Perceived health: EuroQol-5D, EQ-VAS<br>• Usability: User Experience Questionnaire, System Usability Scale (SUS), Technology Acceptance Model		• EG showed significant improvements in QOL (p=.007), depression levels (p=.03), but no significant change in anxiety levels.<br>• 50% reduction in nicotine use in EG.<br><IHD group><br>• EG showed significant improvements in VO_2max (p<.001); CG decreased slightly.<br>• Depression improved markedly in EG; anxiety stable.<br>• EG showed significant improvements in QOL; CG unchanged.<br>• User experience was rated positively. Patients reported that the system was user-friendly, and acceptance of the system was rated highly (mean score > 68).
Caballero et al. (2025) / Brazil [33]	To explore experiences, motivation, and physical outcomes of HF patients participating in VR–based mobilization program during ICU admission	RCT, mixed-methods study	No	60 (30/30)	2	44:16 (73.3:26.7)	59.7±12.2	• EG: Early mobilization with immersive VR (VR glasses showing interactive 360° video), included 3 staged exercises using a cycle-ergometer for upper/lower limbs, sitting/standing, and walking, 10-20 min/day, during ICU stay<br>• CG: Mobilization without VR	• Mobilization experience: Net Promoter Score, open-ended interviews<br>• Dyspnea: Borg Dyspnea Scale	• Baseline<br>• Post (immediate after intervention)	• Mobilization experience was rated “Good/Excellent” in 90% overall; EG 93.3% vs CG 86.7%; between-group differences not significant.<br>• Dyspnea reported better/same post-session overall (EG 53.3% vs CG 50%).<br>• In qualitative analysis, EG reports more pronounced psychological benefits. Physical performance improvements observed in both groups, with emphasis on EG. “Innovation” emerged only in EG.
Costa et al. (2025)	To assess usability	One group	No	10	0	6:4 (60:40)	55.5±11.3	Immersive VR-assisted exercise:	• Enjoyment of exercise: Physical	• Post (immediate	• Exercise enjoyment scored 79.6 (high enjoyment).

/ Brazil [34]	and enjoyment of PA associated with immersive VR among hospitalized patients with HF	pilot study						Patients performed seated lower-limb cycling using a portable cycle ergometer synchronized with VR environment provided by VZFit app via headset. Each session guided active pedaling without load for a comfortable duration, with continuous hemodynamic monitoring.	Activity Enjoyment Scale<br>• Anxiety and depression: HADS<br>• System usability: SUS	after intervention)	• Patients favorably assessed system's usability, with mean score of 68.2 (interpreted as good usability).<br>• No significant correlations were found between anxiety and depression profiles and usability or exercise enjoyment.<br>• Feasibility & safety: No adverse events or cybersickness reported.
Klompstra et al. (2025) / Sweden [29]	To explore effects of exergaming (HF-Wii) and medical yoga on exercise capacity, fatigue, shortness of breath, QOL, depression, and anxiety in patients with HF	Sub-study of RCT	No	104 (EG/Medical yoga group/ CG=35 /33/36)	11 (7/4/0)	66:38 (63:37)	71±12	• EG: Home-based Nintendo Wii Sports (bowling, boxing, golf, tennis, baseball), 30 min/day, 5 days/week, for 12 weeks, telephone calls at 2, 4, 8, 12 weeks<br>• Medical yoga group: 60-min group sessions twice/week for 12 weeks, plus daily home practice, telephone calls at 2, 4, 8, 12 weeks<br>• Control group: Standard PA advice and motivational support	• Exercise capacity: 6MWT<br>• Symptoms: Numeric Rating Scale for fatigue and shortness of breath<br>• QOL: MLHFQ<br>• Well-being: Cantril's Ladder of Life<br>• Anxiety and depression: HADS	• Baseline<br>• Post 1 (3 months)<br>• Post 2 (6 months)<br>• Post 3 (1 year)	• EG improved 6MWT at 3 and 6 months (p<.01); reduced fatigue (p=.043) and shortness of breath up to 6 months; improved physical QOL at 3-6 months (p<.05); but effects diminished by 12 months. There was no significance in well-being or depression and anxiety.<br>• Medical yoga group improved 6MWT at 12 months (p=.049); improved fatigue at 3-6 months; improved emotional QOL sustained at 12 months.<br>• CG showed no significant improvement; well-being declined at 3 months (p=.047).
Andreass	To assess	One	No	14	0	9:5	72	Indoor and	• Daily walking	• Baseline	• Manageable and achievable daily

en et al. (2025) / Multinational (Sweden, Spain) [35]	feasibility of a mobile exergame (Heart Farming) for patients with HF	group pilot study				(64:36)		outdoor mobile exergame (Heart Farming): walking +10 min/day, personalized goals from 6MWT for 4weeks	distance: Heart Farming Exergame Data (In-app tracking)<br>• Implementation, demand, practicality, acceptability: open-ended interview	• Post (1 month)	walking targets (mean daily walking distance 704m, range 250–900m)<br>• The exergame was perceived as easy to learn and use, and adaptable to individual needs.<br>• The intervention was perceived as relevant and needed, and the exergame was considered suitable for reducing sedentary behavior reporting improved physical health.<br>• Participants found the farm theme appealing while the coaching encouraged regular performance of the exergame.<br>• No major acceptability issues were identified.
