# Supplementary material for: Effects of Virtual Reality–Based Interventions for Promoting Physical Activity in Patients With Heart Failure: Systematic Review
Source: J Med Internet Res. 2026 Mar 24;28:e86567. doi: 10.2196/86567 (PMC13012233; doi:10.2196/86567)
Supplement: Checklist 1 [file jmir-v28-e86567-s003.pdf]

PRISMA 2020 Expanded Checklist

No.	PRISMA Item	Requirement	Location in Manuscript	How Addressed
1	Title	Identify report as systematic review.	Title page, pp.1	Title clearly states ‘ Effects of Virtual Reality-Based Interventions for Promoting Physical Activity in Patients with Heart Failure: A Systematic Review’
2	Abstract	Provide structured abstract.	Abstract, pp.1	The abstract adequately addresses all key items outlined in the PRISMA 2020 for Abstracts checklist, including the background, objectives, methods, results, and conclusions.
3	Rationale	Describe the rationale for the review in the context of existing knowledge.	Introduction (pp.2-4)	The Introduction adequately describes the rationale for this systematic review by summarizing existing knowledge, identifying gaps in evidence regarding VR-based interventions in patients with heart failure, and justifying the need for the review.
4	Objectives	Provide explicit objectives.	Introduction,pp.4	The objectives of the review are clearly stated.
5	Eligibility criteria	Specify inclusion/exclusion criteria.	Methods – Search Strategy pp.5,6	Eligibility criteria and synthesis methods are clearly described in the Methods section, with minor revisions to the original text to clarify inclusion and exclusion criteria, publication restrictions, and narrative synthesis approaches.
6	Information sources	List all sources and search dates.	Methods – Search Strategy, pp.5	PubMed, CINAHL, Embase, Scopus searched; 10-year limit.
7	Search strategy	Provide full search strategy.	Supplementary File pp.6	Full strategies described; MeSH + keywords.

8	Selection process	Describe study selection process.	Methods – Screening process	The Methods section clearly describes the study selection process, including independent screening by multiple reviewers and resolution of disagreements without the use of automation tools.
9	Data collection process	Describe data extraction.	Methods – Data extraction, pp.6	The data collection process is clearly described, with independent data extraction by two reviewers and resolution of discrepancies through discussion or consultation with a third reviewer.
10a	Data items	List variables sought.	Methods, pp5	Data items (outcomes) are fully reported in accordance with PRISMA 2020 recommendations.
10b	Data items (other variables)	List and define all other variables for which data were sought	Methods, pp.5	All outcome domains were predefined and systematically extracted across reported measures and time points, with physical activity–related outcomes prioritized for synthesis and interpretation.
11	Risk of bias	Describe RoB assessment methods.	Methods – Risk of Bias Assessment, pp.7	Risk of bias assessment methods are adequately reported in accordance with PRISMA 2020 recommendations.
12	Effect measures	Specify effect measures.	Methods	Effect measures are appropriately reported in accordance with the narrative synthesis approach.
13a	Synthesis methods	Describe synthesis methods.	Methods – Synthesis methods pp.8	Eligibility for synthesis was determined using predefined criteria and clearly described in the Methods section.
13b	Synthesis methods	preparing for synthesis	Methods – Synthesis methods pp.8	No data transformations were required, and results were synthesized using reported data without imputation or conversion.

13c	Synthesis methods	tabulation and graphical methods	Methods – Synthesis methods pp.8	Results were presented using structured tables and graphical summaries to display study characteristics, outcomes, and risk of bias.
13d	Synthesis methods	statistical synthesis methods	Methods – Synthesis methods pp.8	A narrative synthesis was conducted, as meta-analysis was not appropriate due to heterogeneity across studies.
13e	Synthesis methods	Synthesis methods – methods to explore heterogeneity	Methods – Synthesis methods pp.8	Potential sources of heterogeneity were explored descriptively, as statistical methods such as subgroup analysis or meta-regression were not applicable.
13f	Synthesis methods	Sensitivity analyses		No sensitivity analyses were conducted, as statistical meta-analysis was not performed.
14	Reporting bias assessment	Present RoB results.	Results (pp.7)+ Figures 2,3	RoB summary for all included studies.
15	Certainty assessment			Certainty of evidence was not formally assessed because a narrative synthesis was conducted without quantitative pooling.
16a	Study selection	Report numbers of studies; flow of studies	Results + Figure 1	The results of the search and study selection process are reported using a PRISMA flow diagram, detailing the numbers of records identified, screened, excluded, assessed for eligibility, and included in the review.
16b	Study selection –	excluded studies		Studies excluded after full-text review and the primary reasons for exclusion are reported in the PRISMA flow diagram.
17	Study characteristics	Present characteristics of included studies.	Results + Table 1	The characteristics of each included study are presented in Table 1.

18	Risk of bias in studies			Risk of bias assessments for each included study are presented in Figure 2.
19	Results of individual studies	Provide results for each study.		Results of individual studies are presented in the Results section and detailed in (Supplementary) Table 1.
20a	Results of synthesis	characteristics & risk of bias	Results pp.8-10	The characteristics and risk of bias of studies contributing to each synthesis are summarized in the Results section and Figure 2.
20b	Results of synthesis	Results of statistical syntheses		Not applicable. No statistical synthesis or meta-analysis was conducted due to heterogeneity across studies.
20c	Results of synthesis	Results of investigations of heterogeneity	Results pp.8-10	Potential sources of heterogeneity were explored descriptively by comparing study characteristics, intervention types, and settings across studies.
20d	Results of synthesis	Results of sensitivity analyses		Not applicable. Sensitivity analyses were not conducted, as no statistical synthesis was performed.
21	Reporting biases			Not applicable.
22	Certainty of evidence			Not applicable.
23a	Discussion	Interpretation	Discussion pp. 20–24	The results are interpreted in the context of existing evidence.
23b	Discussion	Limitations of evidence	Discussion pp. 26,27	Limitations of the included evidence are discussed.
23c	Discussion	Limitations of review processes	Discussion pp. 26,27	Limitations of the review process are described.

23d	Discussion	Implications	Discussion pp. 24–26	Implications for clinical practice and future research are discussed.
24a	Registration			This systematic review was not registered.
24b	Protocol			A review protocol was not prepared.
24c	Amendments			Not applicable.
25	Support			Non-financial support was declared during the submission process.
26	Competing interests			The authors declare no competing interests.
27	Availability of data, code, and other materials			All data used in this review were extracted from published studies; no new datasets or analytic code were generated.
